# Supplementary material for: Impact of Heavy Metals on Cold Acclimation of Salix viminalis Roots
Source: Int J Mol Sci. 2024 Jan 26;25(3):1545. doi: 10.3390/ijms25031545 (PMC10855682; doi:10.3390/ijms25031545)
Supplement: Supplementary file 1 [file ijms-25-01545-s001.zip › supplementary_figures/Supp_Figure S3.GOE-proteo-clust3.pdf]

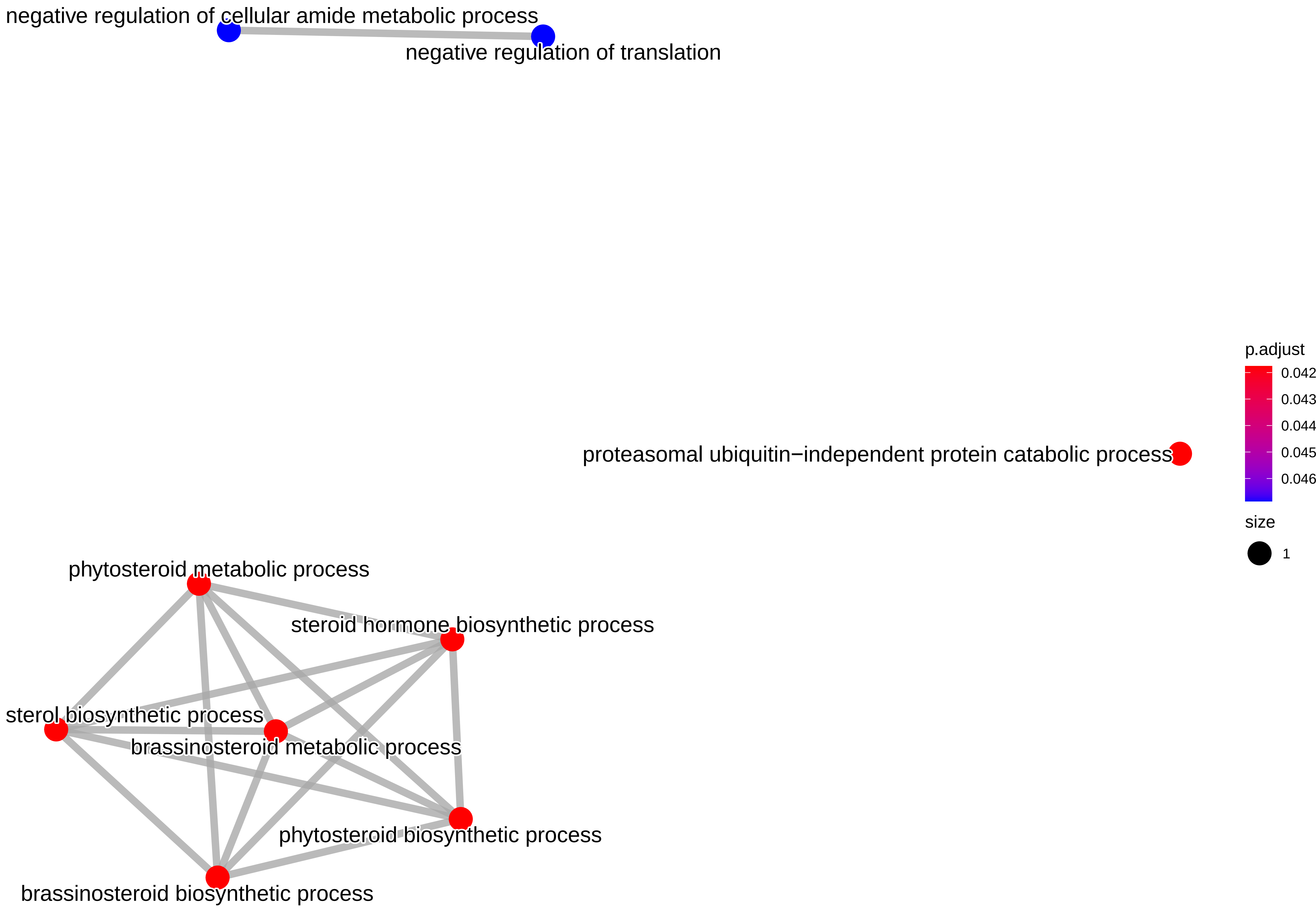
negative regulation of cellular amide metabolic process
negative regulation of translation
proteasomal ubiquitin−independent protein catabolic process
phytosteroid metabolic process
steroid hormone biosynthetic process
sterol biosynthetic process
brassinosteroid metabolic process
phytosteroid biosynthetic process
brassinosteroid biosynthetic process
p.adjust
0.042
0.043
0.044
0.045
0.046
size
1
