# Supplementary material for: Impact of Heavy Metals on Cold Acclimation of Salix viminalis Roots
Source: Int J Mol Sci. 2024 Jan 26;25(3):1545. doi: 10.3390/ijms25031545 (PMC10855682; doi:10.3390/ijms25031545)
Supplement: Supplementary file 1 [file ijms-25-01545-s001.zip › Supplementary_MaterialMethod-Transcriptomicsproteomics-remove ref.pdf]

1. *De novo* transcriptome assembly

1.1. Material and Methods

1.1.1. RNA extraction

Root samples (250 mg) were ground into a fine powder using mortar and pestle in liquid nitrogen. Total RNA was extracted following a modified CTAB buffer extraction protocol [1] and cleaned using the RNeasy plant mini kit (Qiagen, Leusden, The Netherlands) according to the manufacturer's instructions, using an extra DNAse I treatment to remove genomic DNA. RNA purity and quantity were assessed by measuring the absorbance at 230, 260 and 280 nm using a Nanodrop ND1000 spectrophotometer (Thermo Scientific, Villebon-Sur-Yvette, France). Total RNA integrity was assessed using the RNA Nano 6000 assay (Agilent Technologies, Diegem, Belgium) and a 2100 Bioanalyzer (Agilent Technologies, Santa Clara, CA, USA). All RNAs had RIN above 7.

1.1.2. Library preparation and sequencing

Libraries were prepared using SMARTer Stranded RNA-Seq kit (Takara Bio Inc., Mountain View, CA, USA) following the manufacturer's instructions. Briefly, mRNA was isolated from 5 µg of total RNA using Magnosphere UltraPure mRNA Purification Kit (Takara Bio Inc.). The quality of the purified mRNA was assessed using RNA 6000 Pico assay (Agilent Technologies) and a 2100 Bioanalyzer. After purification, mRNA was sheared into fragments of approximately 300 bp, reverse-transcribed into cDNA using random primers, and ligated to Illumina Indexing Primer Set (Takara Bio Inc.), corresponding to Illumina TruSeq indexes 1 to 12. An enrichment step was carried out using 13 cycles of PCR. The samples' average fragment size was determined using a High Sensitivity DNA assay (Agilent Technologies) and a 2100 Bioanalyzer. The pooled libraries were sequenced on an Illumina NextSeq500 (Illumina NextSeq 500/550 High Output Kit v2.5 (300 cycles)) to generate 150 base-pairs pair-end reads. Raw sequences have been deposited at Gene Expression omnibus as accession GSE218490.

1.1.3. *De novo* transcriptome assembly, functional annotation and mapping

Generated FASTQ files were imported in CLC Genomics Workbench v.11.0.1. Sequences were filtered using the following criteria: sequence quality score < 0.01, no ambiguity in the sequence and sequence minimum length of 35 bases. The sequences were trimmed to remove Illumina TruSeq adaptors, poly-A and poly-G sequences, and hard trimmed 8 bases at the 5' end and 3 bases at the 3' end.

De novo assembly was performed using CLC genomics Workbench (version 11.0.1) and Trinity. *De novo* assembly with CLC genomics Workbench was performed in two steps. First, word size was optimised. Assemblies with word sizes ranging from 21 to 61 by 5 increments, an auto bubble size of 50 and a minimum contig length of 300 bp were performed. Word size of 51 gave the best results. Secondly, bubble size was optimised by keeping word size fixed to 51, minimum contig length of 300 and varying bubble size from 50 to 1000 (Supplementary table). An optimal *de novo* assembly was obtained with a bubble size of 500.

De novo assembly was performed twice with Trinity [2,3], using the version integrated into Blast2GO PRO (v. 5.2.5) with the following parameters: paired-end reads, strand-specific forward, pairs distance of 100 and 200 bases.

The three assemblies were merged, and cd-hit-est [4,5], v. 4.7; with parameters -c 1 -n 11) was performed to remove redundant contigs. After that, TransDecoder (v. 5.5.0, https://github.com/TransDecoder/TransDecoder) was performed on the non-redundant merged transcripts to extract putative coding sequences (cds) and peptide sequences. Another step of cd-hit-est (with parameters -c 0.95 -n 10) was performed on the cds to obtain a list of non-redundant peptides, using a 95% sequence identity cut-off. The transcripts corresponding to the non-redundant peptides were then extracted.

The thus selected transcripts were blasted against the annotated *S. purpurea* transcriptome (US Department of Energy Joint Genome Institute), obtained from Phytozome v12.1 [6], https://phytozome.jgi.doe.gov/). Top hit sequences were filtered at 10-5.

Finally, filtered reads were mapped against the *de novo* assembled transcriptome using CLC genomics with the following criteria: mismatch cost of 2, insertion and deletion costs of 3, and length and similar fraction of at least 0.8.

1.2. Results

1.2.1. *De novo* transcriptome assembly, functional annotation and mapping

The *de novo* transcriptome of *S. viminalis* was obtained by merging and assembling the reads obtained by sequencing cDNA libraries of fine roots exposed to cold and/or polymetallic mixture.
A total of 607 614 886 of 150-base pair-sequence reads were obtained, with libraries ranging from 40 to 57 million reads. After trimming and filtering, approximately 480 million reads remained.
Optimised assembly parameters with CLC (word size of 51 and bubble size of 500) produced a transcriptome with 96 582 contigs and an N50 of 538 bases (Supplementary Table 3). The two Trinity assemblies contained 390 400 and 390 421 contigs with an N50 of 389 bases. Merging resulted in a total of 877 403 contigs with an N50 of 416. The first redundancy reduction (cd-hit-est -c 1 -n 11) lowered the number of contigs to 578 179 but increased the N50 to 549, indicating that a large number of small contigs were identical. Similarly, TransDecoder further decreased the number of contigs to 315 611 and increased N50 to 669. As shown in Supplementary Table 3, TransDecoder drastically reduced the number of contigs with a length inferior to 300 bases. The last redundancy reduction using cd-hit-est (-c 0.95 -n 10) reduced the number of contigs to 86 843. About 75% (64 173) of the contigs were successfully annotated using S. purpurea as reference. More than 75% of the filtered reads mapped back to the *de novo* assembled transcriptome, indicating a good quality assembly [2]. Between 17 and 20% of the reads mapped to multiple contigs. This large proportion of reads mapped to multiple contigs likely arises from the whole-genome duplication event, known as the "salicoid" duplication, which occurred 58 Mya [7].

1.3. Discussion

Using three merged assemblies as a starting point increased the odds of catching small and low expressed transcripts that might not be systematically assembled. Its effectiveness is illustrated by the observation that about 65% of the merged transcripts were non-redundant. The successive steps of CDS extraction (TransDecoder) and redundancy reduction (cd-hit-est) brought the metrics close to the one found for the version 5.1 of the *S. purpurea* transcriptome. While this does not necessarily indicate a good transcriptome assembly quality, the congruity between our omics results and previous studies supports our *de novo* transcriptome assembly. In addition, comparable GO results were obtained when using the transcriptome of *S. purpurea* to analyse our transcriptomic data and the proteome of *Populus trichocarpa* and *S. brachista* to analyse our proteomic data (results not shown).

2. Proteomics

2.1. Material and Methods

2.1. Protein extraction and quantification

Soluble proteins extraction was done as described previously [8] with small modifications. Briefly, 400 mg of root were ground in liquid nitrogen and the powder obtained suspended in ice-cold 10% TCA in acetone with 0.07% DTT and centrifuged (15 000 g, 15 min, 4°C). The pellets were washed thrice with ice-cold acetone and dried. Dried pellets were resuspended in 0.7 ml SDS buffer (30% sucrose, 2% SDS, 0.1 M Tris-HCl, pH 8.0, 5% 2-mercaptoethanol) and an equal volume of Tris-saturated phenol (pH 8.0). After vortexing and centrifugation (15 000 g, 5 min, room temperature), the upper phase was transferred to a new tube, and 5 volumes of ice-cold 0.1 M ammonium acetate in methanol were added and kept 2h at -20°C. After centrifugation, precipitated proteins were washed twice with ice-cold 0.1 M ammonium acetate in methanol and twice with 80% ice-cold acetone before being dried. Dried samples were then re-solubilised in labelling buffer (7 M urea, 2 M thiourea, 0.5% w/v CHAPS, 30 mM Tris). Protein concentration was determined with the Bradford method [9] using the Bio-Rad protein assay dye reagent concentrate (Bio-Rad) following the manufacturer's instruction for microplate assay using BSA as standard. All samples were kept at -20°C until further use.

2.1.2. LC-MS/MS

Briefly, 20 µg of total proteins were loaded and separated for a short time of migration on 1D gels (12% Bis-Tris, 1 mm x 12 wells, Criterion, Bio-Rad). Gels were stained with Instant Blue (Gentaur BVBA, Kampenhout, Belgium). Each sample was divided into two halves (referred to as bands A-high molecular weight and B-low molecular weight) and cut into 1-2 mm cubes to perform in-gel digestion. Samples were destained, reduced, alkylated and digested overnight at 37°C using trypsin (sequencing mass grade, Promega, Madison, WI, USA) at a final concentration of 5 ng/µl. The peptides were analysed using a NanoLC 425 Eksigent System (Sciex, Foster City, CA, USA) coupled to a TripleTOF 6600 mass spectrometry (Sciex).

Peptides were loaded onto a trap column (C18 acclaim PepMap™, 5 µm, 5 mm × 300 µm, Thermo Scientific, Bremen, Germany) preconcentrated and desalted for 10 min using loading buffer (2% v/v acetonitrile, 0.05% (v/v) trifluoroacetic acid). After this, peptides were separated onto a C18 reverse-phase column (C18 acclaim PepMap™ 100, 3 µm, 100 Å, 5 µm × 15 cm, Thermo Scientific) at a flow rate of 300 nl/min using a linear binary gradient (solvent A: H2O LC-MS 0.1% formic acid (FA); solvent B: acetonitrile, 0.1% formic acid). Peptides were eluted from 3% B to 30% over 60 min, 40% B during 10 min followed by a wash step to 80% B for 10 min. Prior to the next injection the column was re-equilibrated for 20 min at 3% B. A MS survey scan from 300 to 1250 m/z with 250 ms of accumulation time was followed by 30 MS/MS scans (mass range 100–1500 m/z) using the automatically adjusted rolling collision energy voltage system.

2.1.3. Data analysis

Raw data were imported into Progenesis QI for Proteomics data analysis software (v. 4, Nonlinear Dynamics, Waters, Newcastle upon Tyne, UK). Bands A and B were analysed separately as single fractions then combined. Spectra were processed by Mascot (v 2.6.0, Matrix Science, UK) by searching against a custom-built database using our de novo assembled transcriptome. The search parameters were the following: peptide tolerance of 20 ppm, a fragment mass tolerance of 0.5 Da, carbamidomethylation of cysteine as fixed modification and oxidation of methionine, N-terminal protein acetylation and tryptophan to kynurenine as variable modifications. Proteins identified with a confidence of 95% were kept for further analysis. The fractions A and B were subsequently recombined. Proteins were considered significantly different between conditions when: at least 2 significantly identified peptides per protein of which 1 is unique for the identified protein, ANOVA p-value < 0.05 and a fold change > 1.5. Significantly regulated proteins were characterised using clusterProfiler, based on GO terms from the de novo assembled transcriptome.

The mass spectrometry proteomics data have been deposited to the ProteomeXchange Consortium via the PRIDE [10] partner repository with the dataset identifier PXD030968 and 10.6019/PXD030968.

References

1. Legay, S.; Guerriero, G.; Deleruelle, A.; Lateur, M.; Evers, D.; André, C.M.; Hausman, J.F. Apple Russeting as Seen through the RNA-Seq Lens: Strong Alterations in the Exocarp Cell Wall. *Plant Mol. Biol.* **2015**, *88*, 21–40, doi:10.1007/s11103-015-0303-4.
2. Haas, B.J.; Papanicolaou, A.; Yassour, M.; Grabherr, M.; Blood, P.D.; Bowden, J.; Couger, M.B.; Eccles, D.; Li, B.; Lieber, M.; et al. *De Novo* Transcript Sequence Reconstruction from RNA-Seq Using the Trinity Platform for Reference Generation and Analysis. *Nat. Protoc.* **2013**, *8*, 1494–1512, doi:10.1038/nprot.2013.084.
3. Grabherr, M.G.; Haas, B.J.; Yassour, M.; Levin, J.Z.; Thompson, D.A.; Amit, I.; Adiconis, X.; Fan, L.; Raychowdhury, R.; Zeng, Q.; et al. Full-Length Transcriptome Assembly from RNA-Seq Data without a Reference Genome. *Nat. Biotechnol.* **2011**, *29*, 644–652, doi:10.1038/nbt.1883.
4. Li, W.; Godzik, A. Cd-Hit: A Fast Program for Clustering and Comparing Large Sets of Protein or Nucleotide Sequences. *Bioinformatics* **2006**, *22*, 1658–1659, doi:10.1093/bioinformatics/btl158.
5. Fu, L.; Niu, B.; Zhu, Z.; Wu, S.; Li, W. CD-HIT: Accelerated for Clustering the next-Generation Sequencing Data. *Bioinformatics* **2012**, *28*, 3150–3152, doi:10.1093/bioinformatics/bts565.
6. Goodstein, D.M.; Shu, S.; Howson, R.; Neupane, R.; Hayes, R.D.; Fazo, J.; Mitros, T.; Dirks, W.; Hellsten, U.; Putnam, N.; et al. Phytozome: A Comparative Platform for Green Plant Genomics. *Nucleic Acids Res.* **2012**, *40*, 1178–1186, doi:10.1093/nar/gkr944.
7. Dai, X.; Hu, Q.; Cai, Q.; Feng, K.; Ye, N.; Tuskan, G.A.; Milne, R.; Chen, Y.; Wan, Z.; Wang, Z.; et al. The Willow Genome and Divergent Evolution from Poplar after the Common Genome Duplication. *Cell Res.* **2014**, *24*, 1274–1277, doi:10.1038/cr.2014.83.
8. Wang, W.; Scali, M.; Vignani, R.; Spadafora, A.; Sensi, E.; Mazzuca, S.; Cresti, M. Protein Extraction for Two-Dimensional Electrophoresis from Olive Leaf, a Plant Tissue Containing High Levels of Interfering Compounds. *Electrophoresis* **2003**, *24*, 2369–2375, doi:10.1002/elps.200305500.
9. Bradford, M.M. A Rapid and Sensitive Method for the Quantitation of Microgram Quantities of Protein Utilizing the Principle of Protein-Dye Binding. *Anal. Biochem.* **1976**, *72*, 248–254, doi:10.1016/0003-2697(76)90527-3.
10. Perez-Riverol, Y.; Bai, J.; Bandla, C.; García-Seisdedos, D.; Hewapathirana, S.; Kamatchinathan, S.; Kundu, D.J.; Prakash, A.; Frericks-Zipper, A.; Eisenacher, M.; et al. The PRIDE Database Resources in 2022: A Hub for Mass Spectrometry-Based Proteomics Evidences. *Nucleic Acids Res.* **2022**, *50*, D543–D552, doi:10.1093/nar/gkab1038.
