# Supplementary figures and images for: Impact of Heavy Metals on Cold Acclimation of Salix viminalis Roots
Source: Int J Mol Sci. 2024 Jan 26;25(3):1545. doi: 10.3390/ijms25031545 (PMC10855682; doi:10.3390/ijms25031545)

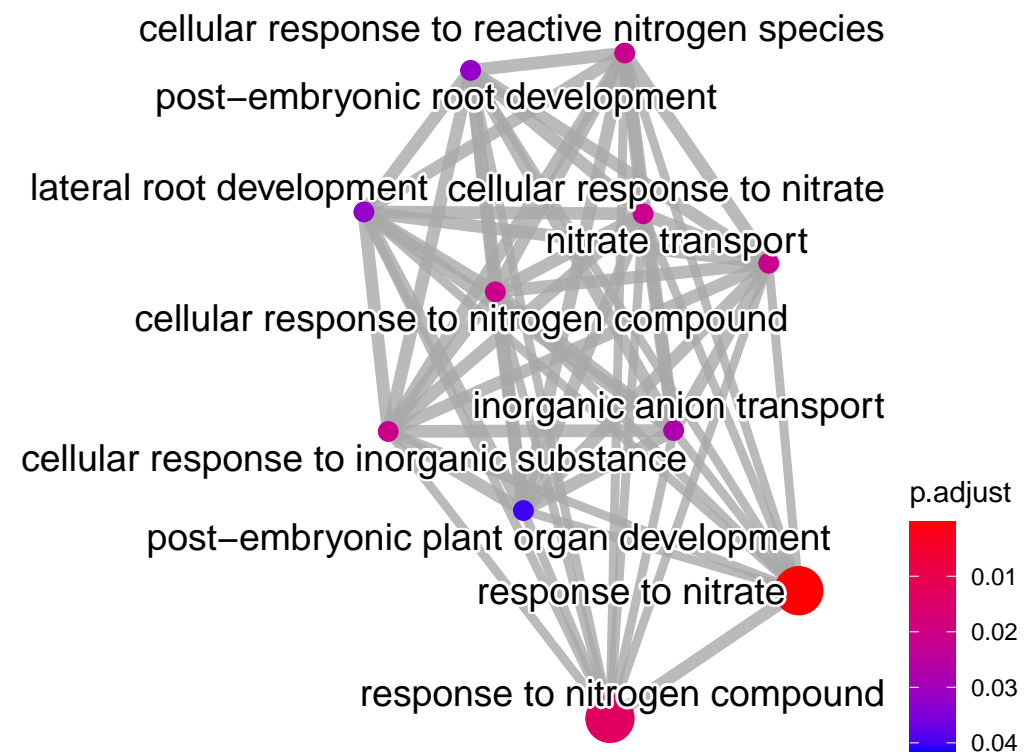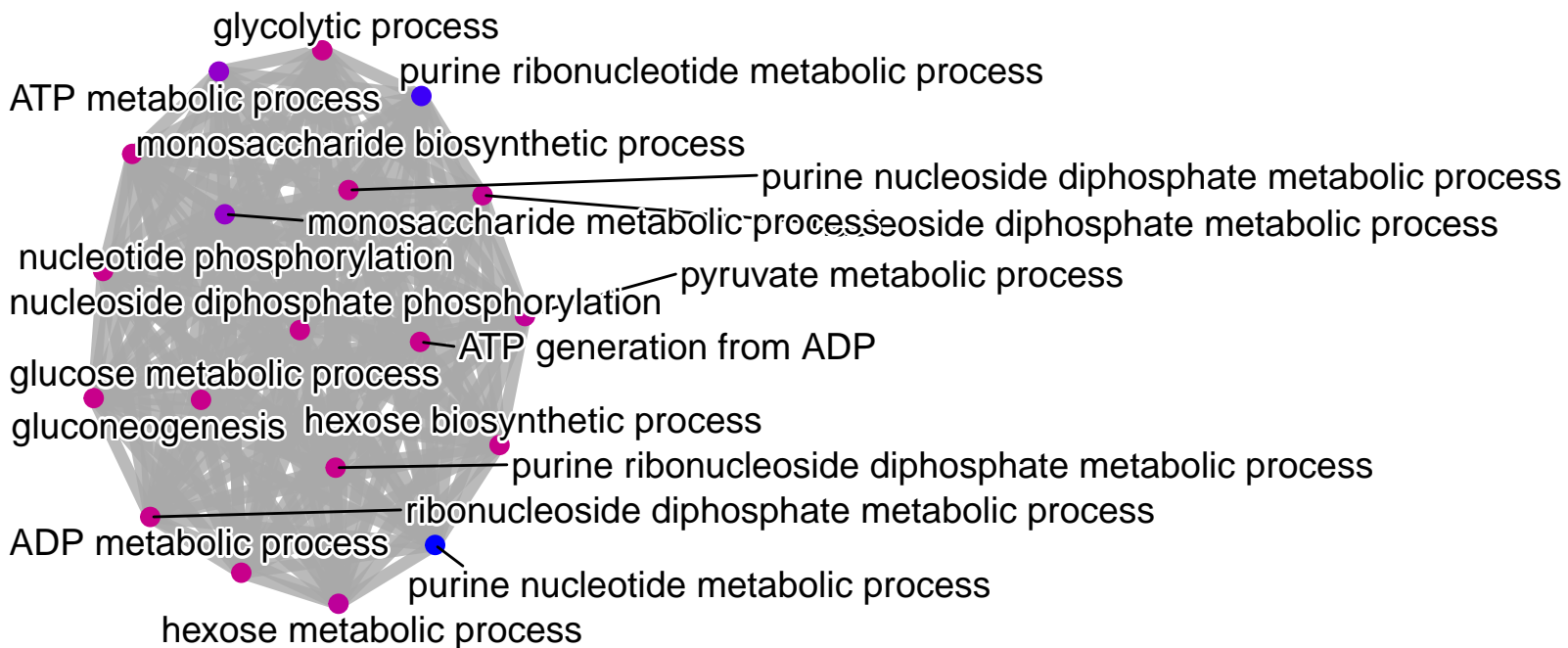

Supplement: Supplementary file 1 [file ijms-25-01545-s001.zip › supplementary_figures/Supp_Figure S1.GOE-proteo-clust1.pdf]

nucleobase-containing small molecule metabolic process

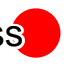

size

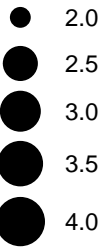

p.adjust

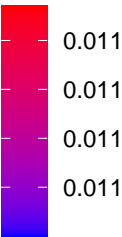

● response to insect

Supplement: Supplementary file 1 [file ijms-25-01545-s001.zip › supplementary_figures/Supp_Figure S2.GOE-proteo-clust2.pdf]

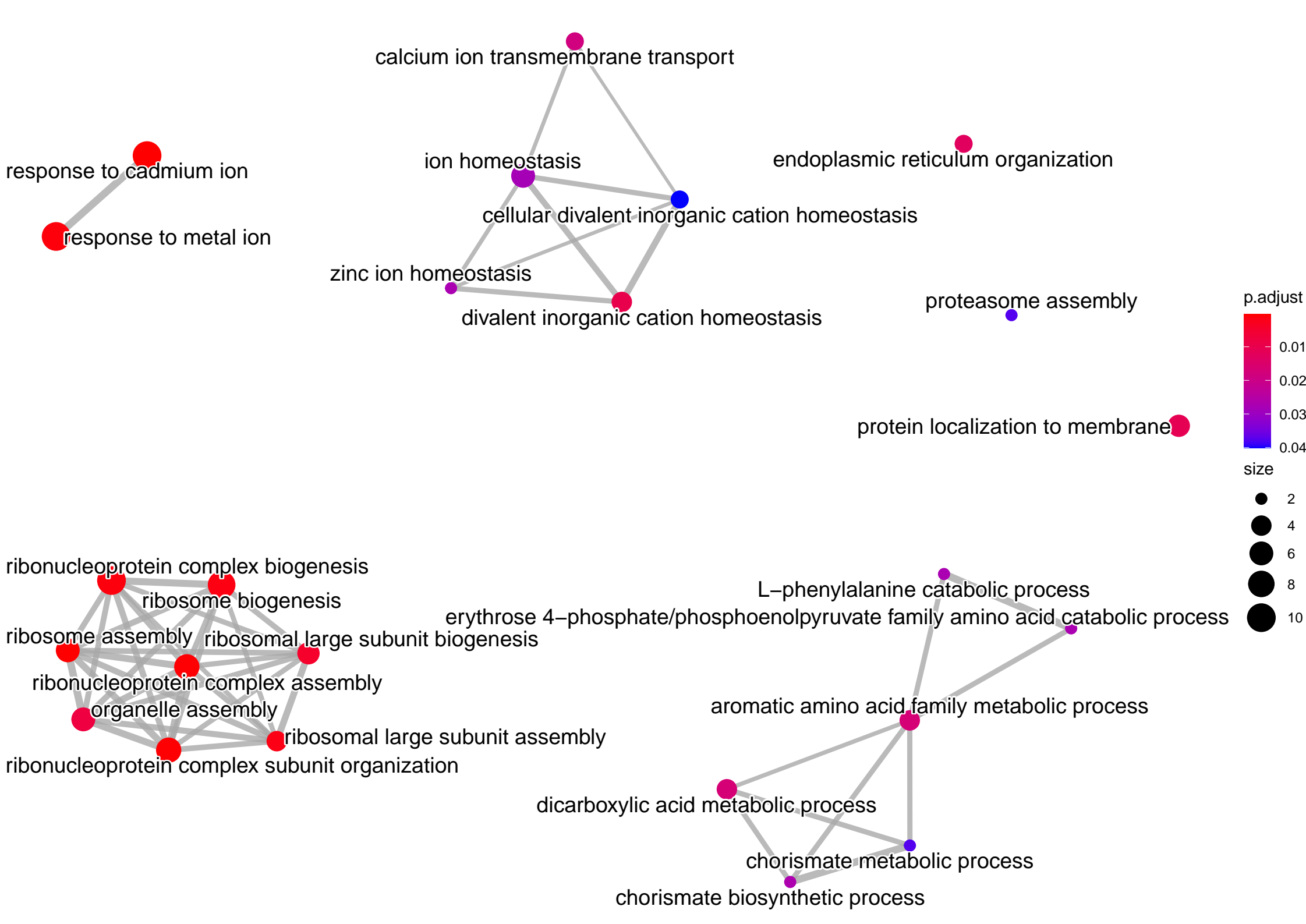

Supplement: Supplementary file 1 [file ijms-25-01545-s001.zip › supplementary_figures/Supp_Figure S4.GOE-proteo-clust4.pdf]

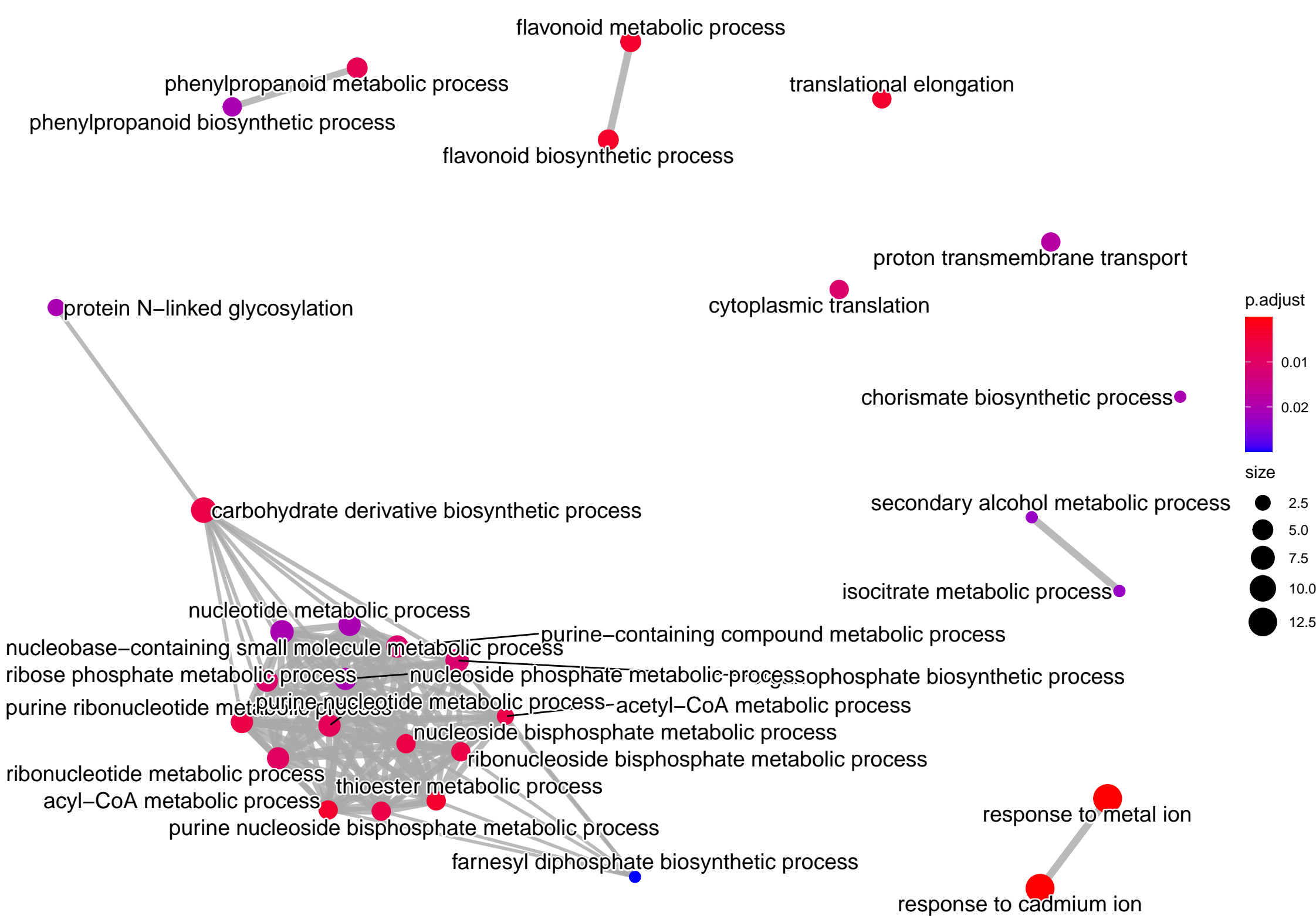

Supplement: Supplementary file 1 [file ijms-25-01545-s001.zip › supplementary_figures/Supp_Figure S5.GOE-proteo-clust5.pdf]

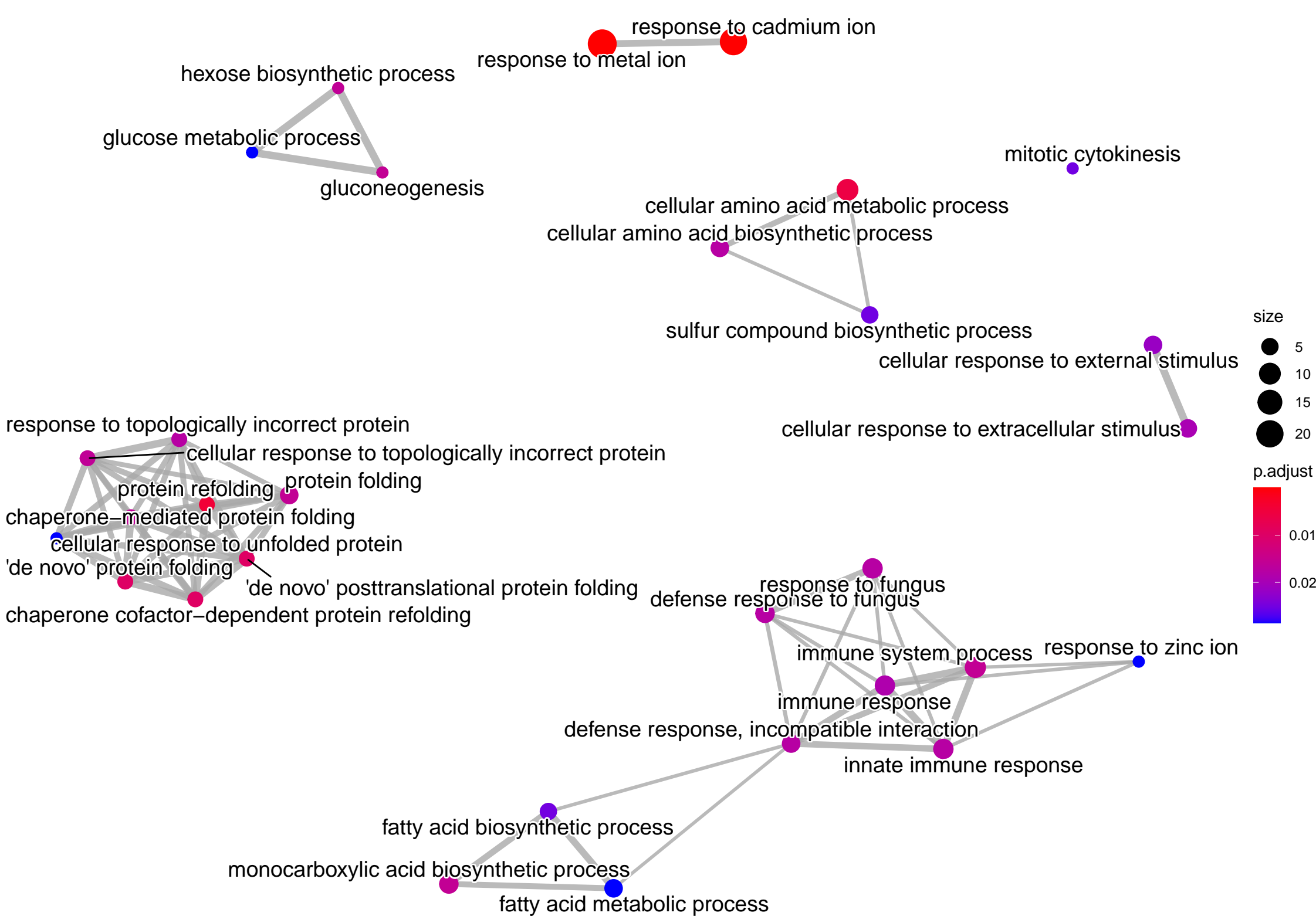

Supplement: Supplementary file 1 [file ijms-25-01545-s001.zip › supplementary_figures/Supp_Figure S6.GOE-proteo-clust6.pdf]

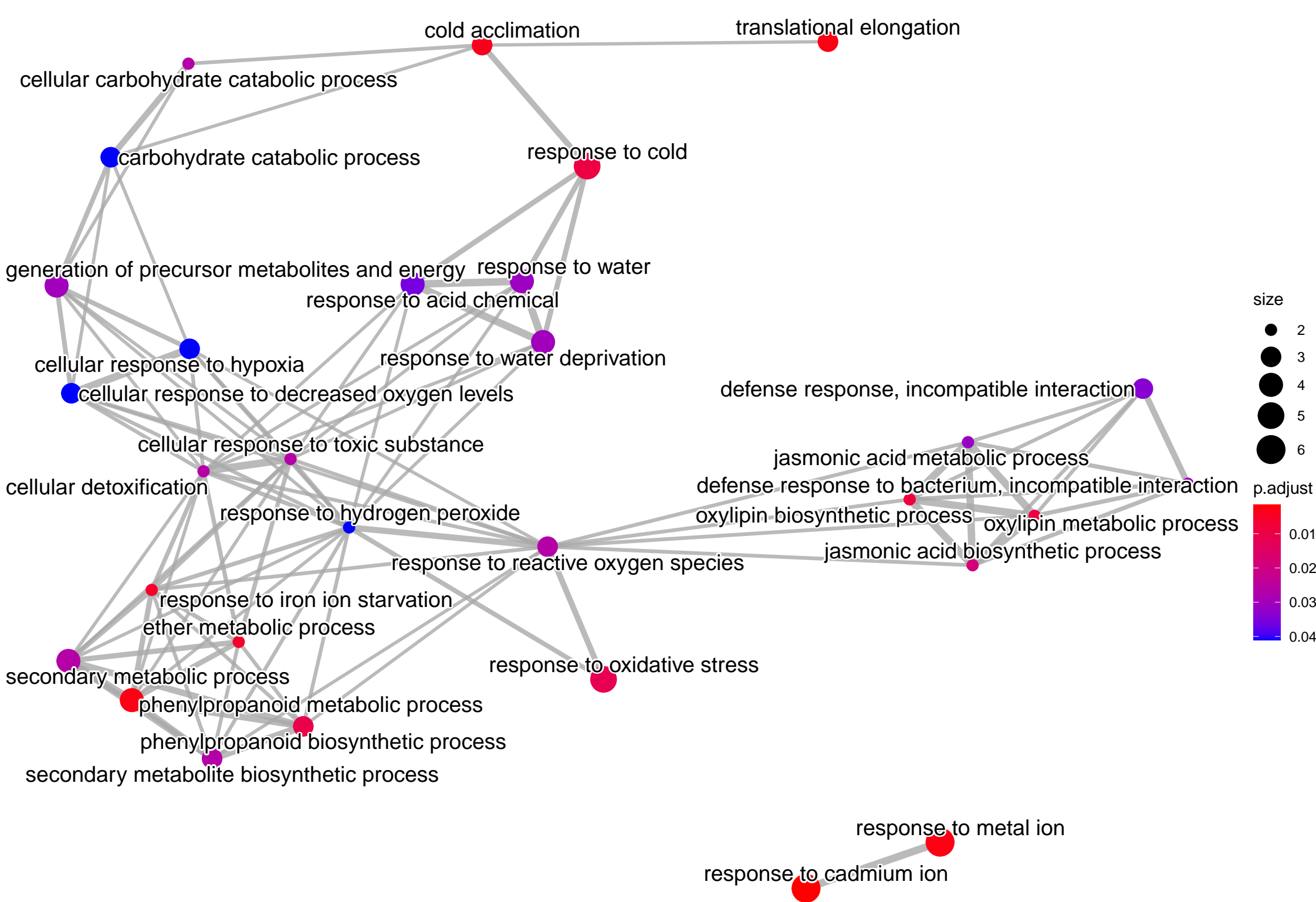

Supplement: Supplementary file 1 [file ijms-25-01545-s001.zip › supplementary_figures/Supp_Figure S7.GOE-proteo-clust7.pdf]
